# Biological determinants of health disparities in multiple myeloma

**DOI:** 10.1038/s41408-018-0118-z

**Published:** 2018-08-28

**Authors:** Cheryl Jacobs Smith, Stefan Ambs, Ola Landgren

**Affiliations:** 1 Laboratory of Human Carcinogenesis, Center for Cancer Research, National Institutes of Health, Bethesda, MD USA; 2 Myeloma Service, Department of Medicine, Memorial Sloan Kettering Cancer Center, New York, NY USA

## Abstract

Multiple myeloma is a rare plasma cell cancer, and incidence rates among patients of African descent are about twice those among patients of European descent. Rates of multiple myeloma vary among different populations, but the reasons for the racial disparities in multiple myeloma are largely unknown. Epidemiology has identified risk factors for multiple myeloma including race, advanced age, gender, family history, and exposure to different genetic toxins including radiation. Race and ancestry play a large role in predicting the risk for multiple myeloma, yet there exists a paucity of literature that explores the molecular contribution of race and ancestry to disease. In this review, we describe the relevant literature that describes the observed racial differences according to distinct tumor immunobiological and ancestral differences in populations.

## Introduction

In whites, multiple myeloma (MM) is the second most common hematological cancer characterized by clonal growth of plasma cells in the bone marrow, producing monoclonal immunoglobulins (M proteins) detectable in peripheral blood and/or in urine^1^. Approximately 30,280 new MM cases (17,490 in men and 12,790 in women) and 12,590 deaths (6660 in men and 5930 in women) are expected to occur in the United States annually^2^. Virtually all MM cases are preceded by the pre-malignant plasma cell disorder monoclonal gammopathy of undetermined significant (MGUS)^3,4^. It is characterized by the presence of M-protein without evidence of MM or other lymphoproliferative malignancies^5,6^.

Among the risk factors for MM including advanced age (≥65 years old), gender (men are more likely to develop MM versus women), family history, radiation exposure, and workplace exposure, racial, and ancestral background play a major role in MGUS and MM incidence^7^. Both disorders are two to threefold more common in African Americans, Afro-Caribbeans, and Africans compared with persons of European ancestry^8–13^. MM is the most common hematological malignancy in African Americans^14^. Moreover, a study conducted to determine the prevalence of MGUS in Ghanaian men versus men of European ancestry observed the prevalence of MGUS in Ghanaian men to be twice that than in men of European ancestry^15^. In contrast, the risk was lower in Japanese and in Mexican Americans^12,16^. The disparity of MGUS and MM between racial and ancestral groups suggests a unique inherited genetic susceptibility and interplay between environmental risk factors.

There is convincing evidence that immune dysregulation plays a major role in lymphoproliferative malignancies such as MM. Investigations have demonstrated that the immune system is quantitatively and qualitatively affected by numerous factors including medical conditions and environmental exposures that can predispose to MM and other lymphoproliferative disorders^11,17–19^. Yet, much less is known about the influence race and ancestry has on immune system dysfunction and the risk of MM.

In this review, we will discuss novel insights concerning the racial disparity of MGUS and MM between persons of African descent and persons of European descent. We focus specifically on the influence of race and ancestry to the cellular immune system and implications for response to treatment.

### Monoclonal gammopathy of undetermined significance and multiple myeloma

Monoclonal gammopathy of undetermined significance (MGUS) is a pre-malignant condition characterized by the presence of a monoclonal immunoglobulin (M-protein) without evidence of MM or a related lymphoproliferative malignancy^5^. Since its description in 1978, the definition of MGUS has evolved^12^. Presently, three distinct clinical MGUS subtypes have been defined: non-IgM MGUS (IgG or IgA), IgM MGUS, and light chain MGUS^20^. Each subtype is characterized by unique intermediate stages and disease outcome. In non-IgM MGUS, the more advanced pre-malignant stage or plasma cell proliferation is smoldering myeloma (10% annual risk of progression to MM; vs. 1% per year collectively for all forms of MGUS)^20,21^. IgM MGUS is associated mainly with Waldenström’s macroglobulinemia and rarely progresses to IgM MM^20^. Light-chain MGUS represents the pre-malignant precursor of “light chain MM,” which accounts for almost 20% of all new MM cases^21^. It should be noted that virtually all MM cases are preceded by a MGUS state, but that most patients with MGUS will not necessarily develop a lymphoproliferative malignancy such as MM^3,4^.

Both MGUS and MM display striking racial and ancestral disparities among persons of African descent compared to persons of European descent^8–13^. The higher incidence of MM in persons of African descent may result from a higher prevalence of the precursor lesion, MGUS, or an increased risk of progression of MGUS to MM in some populations^22,23^. However, definitive studies have observed that the risk of progression from MGUS to MM is the same between racial groups, so the former explanation is more likely^13,15,22,23^.

**Fig. 1:**
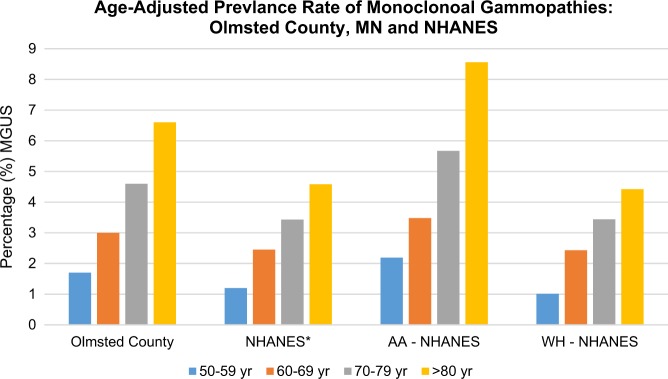
Prevalence of monoclonal gammopathy of undetermined significance (MGUS) in the among residents of Olmsted County, Minnesota compared to persons from the National Health and Nutritional Examination Survey (NHANES). Data has been re-plotted from original publications. Olmsted County, MN study (*n* = 21 462 persons). See reference^25^ for Olmsted County, MN prevalence percentages. NHANES study (*n* = 12 482 persons; *n* = 2331 non-Hispanic blacks [black or African American], *n* = 2475 Mexican Amersicans, *n* = 7051 non-Hispanic whites [white], and *n* = 625 “others”). See ref. ^6^ for NHANES prevalence percentages. Age-adjusted prevalence rates are comparable between studies as the prevalence rates have been similarly standardized to the 2000 US population. Data represents patients over 50 years of age and includes the sexes men and women. AA, African American; WH, Whites. *NHANES age-adjusted prevalence rate contains information from Mexican Americans, whites, and African Americans. However, rates are similar among Mexican Americans and whites. MGUS estimation from the NHANES study likely reflects the true estimate as it is the most representative cohort of the United States population

In a large study assessing the risk of MGUS among African Americans and white veterans in the United States, the study observed that the excess risk of MM in African Americans resulted from an increase risk of MGUS rather than an increased risk of progression from MGUS to MM^23^. Another study estimated the prevalence of MGUS in African Americans and whites utilizing the samples and data from the National Health and Nutritional Examination Survey (NHANES), a nationally representative sample of the United States population^16^. This study observed after screening about 12 400 adults more than 50 years of age that MGUS was significantly higher in African Americans than in whites and Mexican Americans. African Americans not only had a higher overall prevalence of MGUS but also continued to increase with advancing age. African Americans above 80 years of age had a prevalence of 8.6%, nearly double that of whites^16^. However, African Americans were less likely to have IgM MGUS that was associated mainly with Waldenström’s macroglobulinemia and rarely progresses to IgM. These results are consistent with other studies showing lower rates of IgM MGUS in persons of African descent and are in accordance with the racial composition of Waldenström’s macroglobulinemia^13^. When the group studied the prevalence of MGUS in younger individuals aged 10–49 years using samples from NHANES, in persons less than 50 years of age, MGUS was significantly more prevalent in African Americans compared with whites^24^. The estimation of MGUS prevalence from NHANES is more representative of the United States population versus previous MGUS prevalence estimates from the geographically and racially homogenous Olmstead County Study^16,24,25^. The estimation of MGUS prevalence among African Americans, Mexican Americans, and whites from the NHANES study is likely the most accurate estimate to-date (Fig. 1).

Therefore, the racial and ancestral disparity between MGUS and MM among populations of African descent and whites is likely the result of higher incidence of MGUS in persons of African descent. It would be interesting to follow-up on these findings using a large diverse genomic dataset and assay serum samples from African American and whites and analyze their cytokines to determine whether immune factors related to ancestry contribute to the observed MGUS incidence disparity or the disparity observed in MM.

### Ancestry-related differences in disease on-set, response to treatment, and survival

It is well-known that genomic polymorphisms can predispose to disease. Previous reports of familial clustering of MM among first-degree relatives observed a two-fold increased risk of developing MM as well as a two-fold increased risk of MGUS^18^. Thus, first-degree relatives of MGUS patients are at an increased risk of developing both MGUS and MM^18,26^. These observations support a role for inherited susceptibility loci (an interplay of genetics with environmental factors) that predisposes to malignancy. However, the biological impact of inherited susceptibility loci to racial and ancestral disparity of disease has only recently been appreciated due to advancements in genomic technology and the availability of larger, diverse cohorts with sufficient power to detect differences.

A genome-wide association study (GWAS) of MM identified several Human leukocyte antigen (HLA) alleles associated with susceptibility to MM using a novel statistical methodology for fine mapping of HLA associations^27,28^. The study observed that the HLA alleles associated with susceptibility to MM were unique to certain populations. In some instances, HLA alleles that were identified as strong susceptibility loci to MM in whites were not identified to be positively associated to MM in other populations like African Americans, Asian/Pacific Islander, or Hispanic. The study provided evidence that inherited variation in the immune response could increase risk to MM and other mature B-cell malignancies. As well, the study provided additional insight into the difficulty of untangling the impact of environmental interactions with ancestry-related variation.

Hepatitis C virus (HCV) clearance represents a well-known ancestry-related difference in response to treatment among populations of European and African descent. A recent study identified a novel variant that strongly predicts HCV clearance in populations of European, Asian, and African ancestry^29^. One variant, that produces a novel interferon, interferon lambda 4 (IFNL4), was strongly associated with poor clearance of HCV and poor response to treatment. The other “beneficial” INFNL4 variant, a recently derived insertion common to all human populations, was observed to have the lowest frequency in populations of African descent. The unequal frequency of the “beneficial” variant among populations of African descent, in accord with a higher frequency of the variant associated with poor HCV clearance, could explain the differential clearance of HCV and the poor response to HCV therapy along racial and ancestral lines^30^. Future studies should assess the unequal frequency of the “beneficial” INFNL4 variant in additional diverse cohorts with profound disparities in disease to better understand the contribution of the INFNL4 variants to diseases with underlying viral infections or immunobiology disorders such as cancer (e.g., head and neck cancer) or lymphoproliferative disorders like MM.

The relationships between genes, race, and disease are complex and an improved understanding of the unique genetic landscape across patient populations by ancestry and race can potentially identify populations at risk of developing particular diseases and/or that are superior in response to treatment.

### Multiple myeloma disease biology and clinical outcomes

**Fig. 2:**
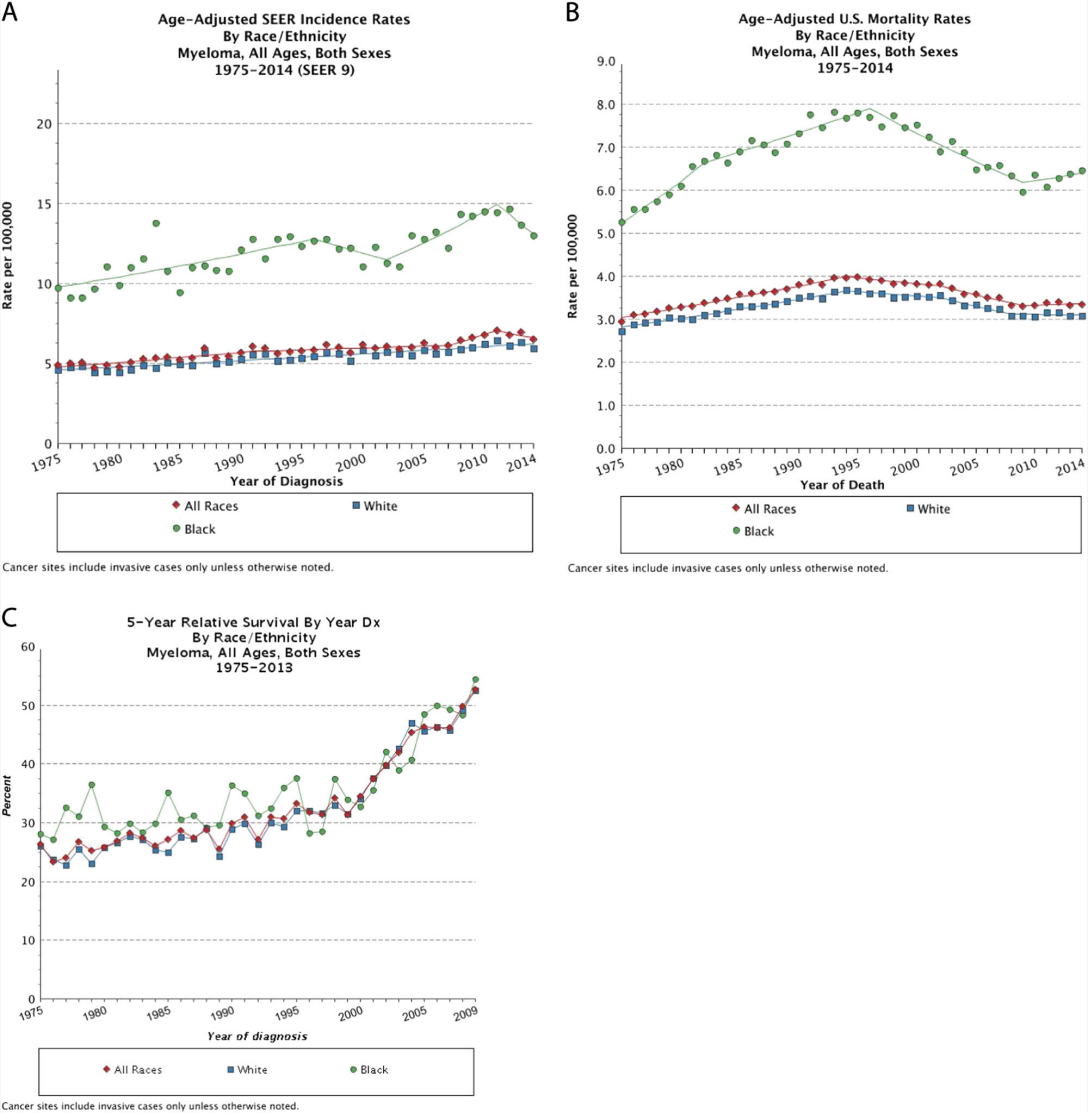
The Yin and Yang of mortality and survival in multiple myeloma. **a**, **b**, **c** Age-adjusted rates of multiple myeloma incidence (**a**), mortality (**b**), and 5-year survival percentae (**c**) in all races (red, diamond), blacks (green, circle), and whites (blue, square) from the Surveillance, Epidemiology, and End Results (SEER) program of the National Cancer Institute (NCI) between 1975 and 2014. Despite the 2–3 times higher incidence and mortality observed among blacks with multiple myeloma, the 5-year survival for blacks is equal to superior to whites given the year

Prior studies have noted distinct differences in patients of African descent with MGUS compared to whites, including lower levels of M-protein, younger average age of distribution, and a lower prevalence of IgM gammopathy^5,24,31,32^. Additionally, African–American patients matched for socioeconomics, age, and gender have two times higher mortality rate than multiple myeloma patients of European descent. Paradoxically, in an analysis of over 30 000 patients with MM in the Surveillance, Epidemiology, and End Results (SEER) registries, African-American patients with MM had better survival as compared to whites^32^. How is this so? It is important to note that although African-American MM patients have higher mortality than white MM patients, it is because mortality is a measure of the frequency of deaths, which is partly dependent on incidence—whereas survival is not (Fig. 2a, b)^33^. Thus, higher mortality is observed among African-American MM patients because MM is more common, regardless of outcome. This is an important distinction when dealing with disparities in MM—although patients of African descent are more likely to develop MGUS and MM as compared to patients of European descent, their disease-specific survival is superior (Fig. 2c)^33^. Additionally, despite the clinical and biological differences among race and ancestry, the rate of transformation of MGUS to MM appears to be similar between patients of African descent and patients of European descent^23^. It is important to remember the clinical context of MM in regards to the disparity—the disparity among patients of African descent is not unilaterally inferior to whites.

Tumor heterogeneity likely may play a large role in the observed disparity in MM. In this regard, a retrospective study assessed the frequency of four cytogenetic abnormalities routinely tested for after diagnosis with MM. The presence of these cytogenetic abnormalities were screened for in 292 African–American MM patients newly diagnosed. In this study, 63.4% of African–American MM patients vs. 34.6% of white MM patients harbored a hyperdiploid or trisomic form of MM which is clinically known to have a better prognosis^34^. Later studies explored the genomic landscape of multiple myeloma tumors among African Americans and whites and observed distinct differences. The genomic landscape of tumors derived from whites were more complex characterized by many more genomic translocations and mutations across the genome when compared to African–American tumors^14^. However, this study lacked clinical data regarding diagnosis and treatment for all patients which may have affected the detection of the genomic abnormalities. This group later followed up with a more comprehensive dataset with the sought after clinical variables. The follow-up study contained high quality whole exome sequencing and RNA-seq from 721 newly diagnosed MM patients who self-identified as either African American (*n* = 128) or white (*n* = 593)^35^. To complete the self-identified race of the participants, the subjects were genotyped for 4761 Ancestry Informative Markers (AIMs) SNPs and population stratification conducted using principal component analysis. This study confirmed previous findings that African Americans demonstrated earlier disease onset of MM yet, a similar rate of overall survival to whites.

Additionally, this study identified gene mutation clusters more frequent among whites. MM tumors from whites were observed to commonly harbor *TP53* mutations^35^. Clearly, more up-to-date advance molecular profiling, such as DNA and RNA sequencing, in the context of race and ancestry, will be important to further clarify the details of these observations.

### The influence of race and ancestry on the immune system

Other cancer-related disparities exist outside of MM between African Americans, Afro-Caribbeans, and Africans as compared to whites. One of the most prominent cancer health disparities exists in prostate cancer. The incidence and mortality rates of prostate cancer are significantly higher in African–American men when compared to whites^2^. Some studies have suggested that prostatitis (inflammation of the prostate gland) may be linked to an increased risk of disease, but this has yet to be fully investigated. However, inflammation is often observed in samples of prostate tissue that also contain cancer^36–39^.

A study using microarray technology obtained gene expression profiles of primary prostate tumors resected from 33 African-American patients and 36 white patients matched on clinical variables. This study revealed a significant number of genes differentially expressed between African–American primary prostate tumors and tumors from white men^36^. Moreover, using a disease association analysis, the group identified a common relationship of these transcripts with autoimmunity and differentially expressed genes clustering in immune response, stress response, cytokine signaling, and chemotaxis pathways. Often, the immune-related genes were more highly expressed in tumors of African-American patients when compared to white patients.

More recently, the reactivation of endogenous retroviruses in the HERV-K family has been associated with prostate cancer diagnosis^40–42^. A study examining aberrant expression of human endogenous retroviruses in prostate cancer identified that aberrant expression of endogenous retroviruses in prostate tumors was significantly higher in African–Americans compared to whites^43^.This difference was also observed among healthy controls where endogenous retroviruses were aberrantly expressed at higher baseline levels in African Americans. An additional study identified that the frequency of allelic variation among the various endogenous retroviruses was greater in African populations than populations from Europe or Asia^44^. Furthermore, others reported significantly higher insertion frequencies of endogenous retroviruses in African Americans when compared to persons of European descent^45^. Because African Americans may have inherited a greater number of polymorphic endogenous retrovirus loci than persons of European descent, this could explain the increase in the number of endogenous retroviral loci transcribed. Prostate tumors isolated from African Americans may have a more “activated” immune system and therefore contribute to increased endogenous retroviral expression in these tumors.

In addition, a recent study observed an inverse relationship with advanced prostate cancer and disease recurrence among African–American men that regularly took aspirin, an anti-inflammatory drug and yet, this relationship was not identified in whites^46^.

Taken together, these studies provide evidence that immune differences between African Americans and whites can contribute to the observed disparity in tumor biology—in this case the increased incidence, morbidity, and mortality observed in prostate cancer among African Americans when compared to whites. Thus, it is conceivable that immune differences may contribute to the disparity and incidence of MM observed in populations of African descent when compared to populations of European descent. Additional research investigating tumor immunobiology differences between populations would provide insight into the role of the immune system in the pathogenesis of cancers with large racial and ancestral disparities.

## Summary and conclusion

While new concepts and treatment strategies have emerged, the increased incidence of MGUS and MM among African Americans persists and remains 2–3 times greater versus persons of European ancestry, Asian (Japanese), and Mexican–American populations^2,16^. The underling mechanisms are not well-understood but there are observations that provide support to the hypotheses surrounding this disparity. Firstly, the observed disparity of MM between patients of African descent and patients of European descent may be due to inherited susceptibility loci. This observation is supported by the study that identified increased polyclonal hypergamma-globulinemia in Ghanaian men versus white men living in the same geographical area^47^. Interestingly, the racial disparity in polyclonal hypergamma-globulinemia parallels the disparity seen in other plasma cell proliferative disorders, such as MGUS and MM. Although, a relationship between polyclonal hypergamma-globulinemia and the development of MGUS has not been fully established, the higher incidence of both conditions in the same population group suggests a possible parallel relationship. Indeed, studies have identified that African Americans harbor variants in genes that are involved in the immune system that could predispose to hematological disease^28,48^.

In one instance, inherited susceptibility alleles influenced the ability of the immune system to properly clear viruses and in another, immunobiology gene enrichment in tumors isolated from African–American men pointed to the contribution of the inflammatory response in prostate cancer disparity. So much so that African–American men who regularly took aspirin were found to have a decreased risk of developing prostate cancer, aggressive disease, and had decreased disease recurrence^46^.

To better understand how race and ancestry contribute to disease we need more diverse cohorts to conduct larger and better powered genomic and epidemiological analyses combining data, biospecimens, and expertise from computational biology, molecular biology, and behavioral sciences. Research also needs to allow for greater research participant participation in research studies. It is increasingly clear that research participants expect that researchers will report back to them how their data was used to produce study results. Greater participant engagement in research studies is likely to incentivize participation in studies across all groups. Ensuring participant protection while also creating a platform that encourages participant engagement in the research process is a key factor in recruiting from historically under-represented populations that, because of the lack of representation, have been left out of many health discoveries.

Efforts such as the Multiple Myeloma Research Foundation (MMRF) CoMMpass Study and the National Institutes of Health All of Us Research Program seek to recruit large, diverse cohorts that allow for greater participant accountability of their data (e.g., digital health monitoring devices, tiered informed consent) and facilitate data sharing to researchers from around the world to enhance innovative medical discoveries. CoMMpass’ commitment to study a wide range of patients with MM throughout the lifetime of a patient’s treatment will give almost instant feedback to researchers and the medical community about what treatments are superior or inferior to certain populations. The CoMMpass study and the All of Us Research Program’s historic effort to gather health (e.g., lifestyle, environment) and biological data from one million or more people in the United States (US) will provide invaluable cohorts to researchers to continue their work in genetic admixture mapping to provide insights into how ancestral alleles contribute to disease disparities. Additionally, the genotype data combined with the rich clinical variables collected over the longitudinal lifetime of study participants will allow researchers to begin to hone in on specific environmental interactions and behaviors that interplay with ancestral genetics that put certain populations at increased risk for disease.

As clinical medicine improves its diagnostic capabilities to detect carcinogenesis earlier and tailor treatment options, the customization of healthcare using familial history, medical history, and ancestry and/or race will likely improve disease prevention and treatment.

## References

[1] Benjamin M, Reddy S, Brawley OW (2003). Myeloma and race: a review of the literature. Cancer Metastas. Rev..

[2] Howlader N. N. A., et al. SEER Cancer Statistics Review, 1975–2014. 2017; based on November 2016 SEER data submission.

[3] Landgren O (2009). Monoclonal gammopathy of undetermined significance (MGUS) consistently precedes multiple myeloma: a prospective study. Blood.

[4] Weiss BM, Abadie J, Verma P, Howard RS, Kuehl WM (2009). A monoclonal gammopathy precedes multiple myeloma in most patients. Blood.

[5] Weiss BM (2011). Patterns of monoclonal immunoglobulins and serum free light chains are significantly different in black compared to white monoclonal gammopathy of undetermined significance (MGUS) patients. Am. J. Hematol..

[6] Kyle RA, Rajkumar SV (2007). Epidemiology of the plasma-cell disorders. Best Pract. Res. Clin. Haematol..

[7] Alexander DD (2007). Multiple myeloma: a review of the epidemiologic literature. Int. J. Cancer.

[8] McFarlane H (1966). Multiple myeloma in Jamaica: a study of 40 cases with special reference to the incidence and laboratory diagnosis. J. Clin. Pathol..

[9] Talerman A (1969). Clinico-pathological study of multiple myeloma in Jamaica. Br. J. Cancer.

[10] McFarlane H, Talerman A, Steinberg AG (1970). Immunoglobulins in Jamaicans and Nigerians with immunogenetic typing of myeloma and lymphoma in Jamaicans. J. Clin. Pathol..

[11] Brown LM, Gridley G, Check D, Landgren O (2008). Risk of multiple myeloma and monoclonal gammopathy of undetermined significance among white and black male United States veterans with prior autoimmune, infectious, inflammatory, and allergic disorders. Blood.

[12] Landgren O, Weiss BM (2009). Patterns of monoclonal gammopathy of undetermined significance and multiple myeloma in various ethnic/racial groups: support for genetic factors in pathogenesis. Leukemia.

[13] Greenberg AJ, Vachon CM, Rajkumar SV (2012). Disparities in the prevalence, pathogenesis and progression of monoclonal gammopathy of undetermined significance and multiple myeloma between blacks and whites. Leukemia.

[14] Baker A (2013). Uncovering the biology of multiple myeloma among African Americans: a comprehensive genomics approach. Blood.

[15] Landgren O (2007). Prevalence of monoclonal gammopathy of undetermined significance among men in Ghana. Mayo Clin. Proc..

[16] Landgren O (2014). Racial disparities in the prevalence of monoclonal gammopathies: a population-based study of 12,482 persons from the National Health and Nutritional Examination Survey. Leukemia.

[17] Soderberg KC, Hagmar L, Schwartzbaum J, Feychting M (2004). Allergic conditions and risk of hematological malignancies in adults: a cohort study. BMC Public Health.

[18] Kristinsson SY (2009). Genetic and immune-related factors in the pathogenesis of lymphoproliferative and plasma cell malignancies. Haematologica.

[19] Dosani T, Carlsten M, Maric I, Landgren O (2015). The cellular immune system in myelomagenesis: NK cells and T cells in the development of MM and their uses in immunotherapies. Blood Cancer J..

[20] Rajkumar SV, Kyle RA, Buadi FK (2010). Advances in the diagnosis, classification, risk stratification, and management of monoclonal gammopathy of undetermined significance: implications for recategorizing disease entities in the presence of evolving scientific evidence. Mayo Clin. Proc..

[21] Zingone A, Kuehl WM (2011). Pathogenesis of monoclonal gammopathy of undetermined significance and progression to multiple myeloma. Semin. Hematol..

[22] Cohen HJ, Crawford J, Rao MK, Pieper CF, Currie MS (1998). Racial differences in the prevalence of monoclonal gammopathy in a community-based sample of the elderly. Am. J. Med..

[23] Landgren O (2006). Risk of monoclonal gammopathy of undetermined significance (MGUS) and subsequent multiple myeloma among African American and white veterans in the United States. Blood.

[24] Landgren O (2017). Prevalence of myeloma precursor state monoclonal gammopathy of undetermined significance in 12372 individuals 10-49 years old: a population-based study from the National Health and Nutrition Examination Survey. Blood Cancer J..

[25] Kyle RA (2006). Prevalence of monoclonal gammopathy of undetermined significance. N. Engl. J. Med..

[26] Jain M, Ascensao J, Schechter GP (2009). Familial myeloma and monoclonal gammopathy: a report of eight African American families. Am. J. Hematol..

[27] Gragert L (2014). Fine-mapping of HLA associations with chronic lymphocytic leukemia in US populations. Blood.

[28] Beksac M (2016). HLA polymorphism and risk of multiple myeloma. Leukemia.

[29] Prokunina-Olsson L (2013). A variant upstream of IFNL3 (IL28B) creating a new interferon gene IFNL4 is associated with impaired clearance of hepatitis C virus. Nat. Genet..

[30] Aka PV (2014). Association of the IFNL4-DeltaG allele with impaired spontaneous clearance of hepatitis C virus. J. Infect. Dis..

[31] Pulte D, Redaniel MT, Brenner H, Jansen L, Jeffreys M (2014). Recent improvement in survival of patients with multiple myeloma: variation by ethnicity. Leuk. Lymphoma.

[32] Waxman AJ (2010). Racial disparities in incidence and outcome in multiple myeloma: a population-based study. Blood.

[33] Fast Stats: An interactive tool for access to SEER cancer statistics.

[34] Greenberg AJ (2015). Racial differences in primary cytogenetic abnormalities in multiple myeloma: a multi-center study. Blood Cancer J..

[35] Manojlovic Z (2017). Comprehensive molecular profiling of 718 Multiple Myelomas reveals significant differences in mutation frequencies between African and European descent cases. PLoS Genet..

[36] Wallace TA (2008). Tumor immunobiological differences in prostate cancer between African-American and European-American men. Cancer Res..

[37] Powell IJ (2013). Genes associated with prostate cancer are differentially expressed in African American and European American men. Cancer Epidemiol Biomark Prev.

[38] Rose AE (2010). Copy number and gene expression differences between African American and Caucasian American prostate cancer. J. Transl. Med..

[39] Hardiman G (2016). Systems analysis of the prostate transcriptome in African-American men compared with European-American men. Pharmacogenomics.

[40] Reis BS (2013). Prostate cancer progression correlates with increased humoral immune response to a human endogenous retrovirus GAG protein. Clin. Cancer Res..

[41] Goering W, Ribarska T, Schulz WA (2011). Selective changes of retroelement expression in human prostate cancer. Carcinogenesis.

[42] Pérot P. et al. Microarray-based identification of individual HERV loci expression: application to biomarker discovery in prostate cancer. J Vis Exp. e50713 (2013). 10.3791/50713 10.3791/50713 PMC3969901 24300377

[43] Wallace TA (2014). Elevated HERV-K mRNA expression in PBMC is associated with a prostate cancer diagnosis particularly in older men and smokers. Carcinogenesis.

[44] Macfarlane C, Simmonds P (2004). Allelic variation of HERV-K(HML-2) endogenous retroviral elements in human populations. J. Mol. Evol..

[45] Jha AR (2009). Cross-sectional dating of novel haplotypes of HERV-K 113 and HERV-K 115 indicate these proviruses originated in Africa before Homo sapiens. Mol. Biol. Evol..

[46] Smith CJ (2017). Aspirin use reduces the risk of aggressive prostate cancer and disease recurrence in African-American men. Oncology.

[47] Buadi F (2011). High prevalence of polyclonal hypergamma-globulinemia in adult males in Ghana, Africa. Am. J. Hematol..

[48] Idelman G (2007). Functional profiling of uncommon VCAM1 promoter polymorphisms prevalent in African American populations. Hum. Mutat..

